# Improving transparency in conservation social science research to enhance quality, equity, and collaboration

**DOI:** 10.1111/cobi.70003

**Published:** 2025-04-01

**Authors:** Marie‐Annick Moreau, Emily Woodhouse

**Affiliations:** ^1^ Department of Anthropology University College London London UK

**Keywords:** conservation ethics, decision‐making, decolonization, interdisciplinarity, positionality, reflexivity, social science methods, ética de la conservación, descolonización, interdisciplinario, métodos de ciencias sociales, posicionalidad, reflexividad, toma de decisiones, 保护伦理, 决策, 去殖民化, 跨学科, 立场, 反思, 社会科学方法

## Abstract

Recognition of the value of multidisciplinary research that bridges natural and social science perspectives has come with calls for conservation scientists to reflect critically on underlying assumptions and power relations involved in the production of knowledge and its application. We propose that improving transparency in conservation social science—around researchers’ positionality, study limitations, and fieldwork challenges—is essential to and depends on enhanced reflexivity and can allow readers to assess research quality, foster ethical research, and support constructive dialogue and collaboration across subdisciplines of conservation science. We assessed gaps and opportunities for enhanced transparency based on an in‐depth review of 39 papers on the social impacts of protected areas published in 12 conservation journals from 2010 to 2022. We evaluated transparency in these publications based on whether authors reported on their collaborations, values, and identity; methodology and methods; data collection; influence of the wider sociopolitical context; potential limitations and challenges; and linked recommendations to evidence. Authors reported consistently on research aims, intended methods, and sampling strategy but provided limited information on their backgrounds; relationships between authors, field teams, and participants; and field site. Gaps included not reporting who collected the data (lacking from 43% of papers), whether data collectors spoke participants’ language (46%), participant recruitment strategy (56%), women's representation in samples (41%), and time spent in the field (28%). Based on our findings, we devised a reflexive tool relevant to field‐based studies and advice on preparing positionality statements for use by researchers, reviewers, and journal editors. We recommend conservation social scientists shift their expectations of what is reflected on and reported in publications, develop positionality statements, engage with other available reflexive tools, and adopt the first person in their writing to make more visible their role and responsibilities in the research process.

## Abstract (es)

Mejoras en la transparencia de las ciencias sociales de la conservación para incrementar la calidad, equidad y colaboración

**Resumen:**

El reconocimiento del valor de la investigación multidisciplinaria que conecta las perspectivas de las ciencias naturales y sociales ha sido acompañado de llamados a los científicos de la conservación para que reflexionen de forma crítica sobre los supuestos subyacentes y las relaciones de poder que intervienen en la producción de conocimientos y su aplicación. Proponemos que la mejora de la transparencia en las ciencias sociales de la conservación ‐en torno a la posición de los investigadores, las limitaciones de los estudios y los retos del trabajo de campo‐ es esencial y depende de una mayor reflexividad, lo que puede permitir a los lectores evaluar la calidad de la investigación, fomentar la investigación ética y apoyar el diálogo constructivo y la colaboración entre las subdisciplinas de las ciencias de la conservación. Evaluamos las brechas y oportunidades para mejorar la transparencia con base en una revisión profunda de 39 artículos sobre los impactos sociales de las áreas protegidas publicados en 12 revistas de conservación entre 2010 y 2022. Evaluamos la transparencia de estas publicaciones en función de si los autores informaban sobre sus colaboraciones, valores e identidad; metodología y métodos; recopilación de datos; influencia del contexto sociopolítico más amplio; posibles limitaciones y retos; y si vinculaban las recomendaciones a las pruebas. Los autores informaron sistemáticamente sobre los objetivos de la investigación, los métodos previstos y la estrategia de muestreo, pero la información sobre sus antecedentes, las relaciones entre los autores, los equipos de campo y los participantes, y el lugar de trabajo fue limitada. Las brechas incluían no informar sobre quién recopilaba los datos (ausente en el 43% de los artículos), si los recopiladores de datos hablaban el idioma de los participantes (46%), la estrategia de reclutamiento de participantes (56%), la representación de las mujeres en las muestras (41%) y el tiempo empleado sobre el terreno (28%). Con base en nuestras conclusiones, creamos una herramienta de reflexión pertinente para los estudios de campo y consejos sobre la preparación de declaraciones de posicionamiento para uso de investigadores, revisores y editores de revistas. Recomendamos a los científicos sociales dedicados a la conservación que cambien sus expectativas sobre lo que se refleja e informa en las publicaciones, que elaboren declaraciones de posicionamiento, que utilicen otras herramientas reflexivas disponibles y que adopten la primera persona en sus escritos para hacer más visibles su papel y sus responsabilidades en el proceso de investigación.

## 提高保护社会科学研究的透明度以提升质量、公平与合作

【摘要】 人们已认识到跨学科研究在沟通自然科学和社会科学观点方面的价值, 并呼吁保护科学家批判性地反思知识产生及其应用所涉及的基本假设和权力关系。我们建议, 围绕研究人员立场、研究局限性和野外工作挑战的问题, 提高保护社会科学的透明度, 这既是增强反思能力的关键, 也有赖于反思能力的提高, 还可以让读者评估研究质量, 推动符合伦理的研究, 以支持保护科学各分支学科之间的建设性对话与合作。本研究深入分析了2010年至2022年间在12个保护期刊上发表的39篇有关保护地社会影响的论文, 并评估了提高透明度的差距和机遇。对这些发表研究透明度的评估是基于作者是否报告了他们的合作关系、价值观和身份, 方法论和研究手段, 数据收集情况, 更广泛的社会政治背景影响, 潜在的局限性和挑战, 以及建议是否与证据相联系。我们发现, 论文一致报告了研究目的、预期方法和采样策略, 但对研究的背景、作者、野外团队和参与者之间的关系, 以及野外研究位点提供的信息却很有限。存在的空缺包括未报告由谁收集数据 (43%的论文) 、数据收集者是否会说参与者的语言 (46%) 、参与者招募策略 (56%) 、样本中的女性比例 (41%) 以及野外研究花费的时间 (28%) 。我们还根据研究结果设计了一种针对野外研究的反思工具, 并为研究人员、审稿人和期刊编辑提供了编写立场声明的建议。我们建议保护社会科学家转变他们对发表论文中反映和报告内容的期望, 制定立场声明, 使用其他已有的反思工具, 并在写作中采用第一人称, 以更清楚地体现他们在研究过程中的角色和责任。**【翻译: 胡怡思; 审校: 聂永刚】**

## INTRODUCTION

Social science approaches are increasingly deployed in conservation to examine anthropogenic drivers of environmental change and the social impacts of interventions (Bennett et al., 2017; Moon et al., 2019). However, there are concerns around data quality and lack of transparent reporting on the underlying assumptions and decisions in interdisciplinary ecological research (Moon et al., 2016; St. John et al., 2014). Weaknesses in study design and data interpretation are worrying given that conservation findings are often applied in the real world, where interventions can have significant impacts on people and nature (Kareiva & Marvier, 2017). At the same time, conservationists and researchers are being urged to recognize the unequal power relationships that underpin the production of knowledge and its application, from the colonial period to the present (Brittain et al., 2020; Redpath et al., 2013; Sandbrook, 2017; Trisos et al., 2021). Reflecting on our own social roles and on the wider sociopolitical and historical dimensions of research is particularly important for conservationists given the sector's long association with colonial histories, racism, oppression, and exclusion (Collins et al., 2021; MacKenzie, 1988).

Perceived lack of transparency on research processes in the conservation literature may reflect many conservationists’ lack of formal training in the social sciences (Archer et al., 2022; Drury et al., 2011; Gardner, 2021) but could also stem from assumptions of what journal editors and readers expect to see in research submissions (Young et al., 2018). Without a clear disciplinary agreement on why reflecting and reporting on the research process matter to conservation science and practice and how to reflect on it, valuable opportunities for achieving more equitable and sustainable conservation outcomes may be lost (Beck et al., 2021).

Greater reflexivity in conservation science is proposed as the basis for more equitable and effective research and practice (e.g., Montana et al., 2020). We contend that transparent reporting in published conservation social science is inherently linked to reflexivity. Without reflexivity, transparency is incomplete and undermined; normalizing transparent reporting will reinforce reflexive practice. Reflexivity is the critical self‐evaluation of a researcher's normative values, social identity, disciplinary perspective, experiences, and emotional responses to participants, for example, as well as the consideration and recognition of how the researcher's positioning may affect the research process and outcomes (Berger, 2015). For conservation scientists, positionality may influence relationships in the field and data interpretation, as well as conservation practice (Beck et al., 2021; Pienkowski et al., 2023). There is a recognized need for proactive and formal adoption of reflexive processes in conservation science (Boyce et al., 2022; Pienkowski et al., 2023), and we propose that improving transparency standards is a vital step in encouraging that shift.

We argue that there are 3 key reasons to enhance transparency in conservation social science publishing: to increase research quality, to promote greater equity in researcher–researched and Global North and Global South research relationships, and to deepen interdisciplinary collaborations. Other conservationists have framed the benefits of transparency as promoting humility and accountability in the face of uncertainty (Stirling & Burgman, 2021) and generating more adaptable and diverse responses (Beck et al., 2021). Like us, these authors contend that enhanced transparency can lead to stronger collaborations for better conservation outcomes. Other scientific fields emphasize the importance of transparency for data quality for the purposes of replication (Bradley et al., 2020; Powers & Hampton, 2019), but transparency is also discussed as a basis for supporting cross‐disciplinary understanding (Marsden, 2020; Tuval‐Mashiach, 2017) and equity, where disclosure of research decisions can avoid harm to participants and the public (Joshi & Bhardwaj, 2018).

We considered the value of transparency for quality, equity, and collaboration in conservation social science and conducted an in‐depth literature review to illustrate how conservation social science researchers have managed transparency in their publications. In essence, herein we ask researchers: how did you get your data?

### Transparency for better research quality

Whether social research involves standardized surveys, interviews, participant observation, visual methods, or other participatory activities, the data and what researchers make of it cannot be fully separated from the researchers’ own interests, values, and characteristics or from the process through which knowledge was produced in interaction with research participants (Descola, 2005). The researcher's influence will be manifest from the inception of a research project in their choice of research question and methodology, how data are collected and analyzed, and how the results are written up and presented. Yet, many conservation researchers continue to hold on to the idea of value‐free objectivity and are reluctant to recognize how their norms and values shape research outcomes (Pascual et al., 2021).

Transparency of research methods and reflexivity to account for subjectivities are key quality criteria in the social sciences, as are other naturalistic approaches, such as triangulation and checking data and interpretations with participants (Guba, 1981). Without reflexivity, transparency can be undermined, compromising the credibility, confirmability, dependability, and transferability of the data, aspects equating to validity and reliability in the natural sciences (Lincoln & Guba, 1985). Transparency around researcher–respondent dynamics in the field is especially important for assessing data quality in the social sciences. Attia and Edge (2017) contend that qualitative research—and we argue any research involving speaking with or observing people—is at heart an empathic pursuit, with the aim of understanding people's lives. Building such knowledge and trust takes time and is influenced by insider–outsider dynamics, aspects of social differentiation, and the wider sociopolitical space. In cross‐cultural research, the use of translators and field assistants adds a further layer of complexity; these individuals bring their own influence to research encounters (MacKenzie, 2016; Twyman et al., 1999). Degrees of relatedness between members of a research team and research participants can be considered on multiple dimensions of positionality, such as age, class, gender, ethnicity, religion, language, and education (Berger, 2015). Although social scientists cannot necessarily control for these characteristics and subjectivities, they can aim to reflect and be transparent in reporting on the most salient ones. This would give readers greater ability to assess the trustworthiness of findings and to determine how applicable research is to other contexts (Moon et al., 2016).

### Transparency for greater equity

Conservation research often takes place in cross‐cultural contexts, frequently across the Global North–Global South divide, where researchers may be very different from the people involved in the research, as in our own experience (see our positionality statement below). Feminist scholarship highlights that issues of positionality and reflexivity are integral to interrogating how research questions, methods, and interpretation may be embedded in unequal power relations between researcher and subject, with implications for knowledge production, representation, and participants’ well‐being (Nast, 1994). Given histories of colonialism, development, and globalization, explicit awareness of one's position is an ethical imperative in order to avoid perpetuating relations of domination and control (Sultana, 2007), not least by reflecting on the less obvious ways one's project could do harm (Guillemin & Gillam, 2004). Increased reflexivity and transparency can open up new possibilities in interaction, bringing more diverse and authentic insights (Attia & Edge, 2017). In particular, acknowledging motivations and values is important for building trust and respectful relationships with a diversity of people, including across Global South–Global North research teams, as a basis for equitable and productive research and action (Boyce et al., 2022; Trisos et al., 2021). With greater transparency, participants, including marginalized groups, such as Indigenous peoples and rural residents of the Global South, are better placed to understand, participate in, and challenge research that may directly affect them (Moon et al., 2016). By explicitly recognizing and leveraging their privilege while critically examining the wider institutional, social, and political context, researchers could also work with participants to achieve positive ends (Brittain et al., 2020).

### Transparency for stronger collaborations

Enhancing transparency is an essential step in establishing more constructive interdisciplinary collaborations. The conservation movement is heterogeneous, characterized by a range of values but still somewhat aligned along the natural–social science divide with biological sciences remaining dominant (Montana et al., 2019; Sandbrook et al., 2019). Calls for more constructive cross‐disciplinary dialogue are not new (Brosius, 2006; Chua et al., 2020; Miller et al., 2011; Montana et al., 2019), but improving the quality, tone, and breadth of the debate is ever more essential given the scale of social and ecological problems facing humanity (Holmes et al., 2016; Tallis & Lubchenco, 2014). Productive conversations and appreciation of different subfields will require paying more explicit attention to the many different “cultural lenses” researchers bring to conservation (Peterson et al., 2010). As these authors explain, the lens metaphor encourages researchers to reflect not only on methodology (how one looks) but also ontology (what aspects of reality one looks at) as they are shaped by researchers’ theoretical preferences and individual idiosyncrasies. A lack of understanding between diverse team members can pose major challenges (Beck et al., 2021), and different disciplinary standpoints can result in very different policy recommendations (Pascual et al., 2021).

How far does conservation social science need to go to achieve greater transparency in published conservation social science? We examined the types and levels of information provided by researchers in research articles and sought to identify opportunities to enhance transparency to improve research quality, equity, and collaboration. To do this, we carried out an in‐depth literature review of peer‐reviewed articles based on field research published since 2010 in which authors studied the links between protected area interventions and human well‐being in low‐ and lower‐middle income countries. We chose this topic because it is particularly contested, has different evidence bases, and often presents a divide between different types of methods used—from ethnographic to more structured and quasi‐experimental (Brockington & Wilkie, 2015). It is also a topic for which the research produced has significant implications for equity. We did not assess the quality of the sampled research papers; rather, we assessed the degree and nature of transparency in how authors reported on their research decisions, assumptions, and challenges.

### Positionality statement

We completed undergraduate degrees in biology and worked in conservation and sustainable development before conducting doctoral research and pursuing careers in anthropology. As members of UCL Anthropology's Human Ecology Research Group, we promote the use of interdisciplinary approaches and mixed methods in our research and teaching. This article was motivated by our personal experience of moving from the realism and objectivism of our natural sciences training toward an ontology and epistemology of critical realism and constructivism and wanting to support others in recognizing how much of the research process is kept hidden from participants, readers, and oneself. We recognize that we have lacked transparency in our earlier publications and are engaged in this learning process. Our education at elite Global North institutions, access to grants, and passport privileges (E.W. as a British citizen, M.‐A.M. as a Canadian citizen) have given us opportunities to conduct extended fieldwork in the Global South, and our relationships in the field are influenced by our identities as White women. We offer our insights as foreigners to our field sites to the discussion of the importance of transparency in the researcher–researched dynamic and acknowledge that the latter's voices are not heard enough.

## METHODS

To find candidate papers to review, we drew on a database of English‐language, peer‐reviewed articles published from 2010 to 2017 on the linkages between area‐based conservation interventions and human well‐being in terrestrial and marine ecosystems in low‐ and lower‐middle income countries (Woodhouse et al., 2022), as classified by the World Bank in 2017 (countries listed by category in Woodhouse et al.’s [2022] supporting information). Second, we conducted a literature search for more recently published (2018–2022) articles on the same topic. We searched in Web of Science (Appendix S1) and used Woodhouse et al.’s (2022) search terms. We restricted the sample to a set of 11 conservation journals accepting social science articles and having an impact factor >2, as determined by Moon et al. (2016). For 2021–2022, we added the new journal *People and Nature* to our database, given its relevance to conservation social science.

Using a random number generated in Excel, we randomly selected for review 3 journal articles per year from among the final list of 185 candidate papers (Appendix S2). Nine papers were excluded on the basis that the work was not based on primary data (e.g., reviews), the work was not related to area‐based conservation interventions (e.g., existing sacred groves, payment for ecosystem services), or authors considered attitudes but not well‐being impacts. We randomly selected nine replacement papers, matched on publication year, to arrive at a sample of 39 articles (Appendix S3).

This sample size provides a snapshot of the state of transparency reporting across an important but specific area of conservation social science and allowed us to engage in depth with each paper to extract high‐quality data (https://doi.org/10.6084/m9.figshare.25722828.v2). We started our review at 2010 to reflect the gathering impetus for incorporating social science into conservation research that began around the turn of the century (Mascia et al., 2003).

We developed a set of criteria to assess transparency in reporting: authors’ collaborations, values, and identity; methodology and methods; data collection and the research team's relationships in the field; influence of the wider sociopolitical context; potential limitations and challenges; and basis for recommendations (Appendix S4). Our set of transparency criteria built on existing guidelines and checklists for improving methodological reporting in conservation social science research (Moon et al., 2016; Young et al., 2018) and others proposed for social science more generally. We supplemented these with criteria relevant to cross‐cultural fieldwork and identified in the literature as having implications for research quality, equity, and collaboration.

Criteria were captured in a Microsoft Form and used to extract data from the papers, following a codebook (Appendix S5). We each reviewed half of the papers including supplementary material. On checking intercoder reliability (mean percent difference in scores 13%, *n* = 4 papers), we revised the codebook on problematic criteria, recoded all the papers accordingly, and checked in with one another wherever doubts arose. Discrepancies arose primarily around each reviewer's perception of what an author had reported on an item. For example, we came to agree that the use of research assistants could not be established from the acknowledgments section alone.

To check for sample saturation, we excluded the 4 most recently added papers from our data set (10% of the sample) and re‐ran our analyses. Percentage scores on our transparency criteria differed little between the 2 sample sets (median difference = 1.4%, range: 0–6%, *n* = 38 transparency criteria), suggesting our sample size was adequate for our purposes. We also checked the trend in transparent reporting by publication year and found no significant change through time in the number of criteria reported on (Appendix S6).

## RESULTS

**FIGURE 1:**
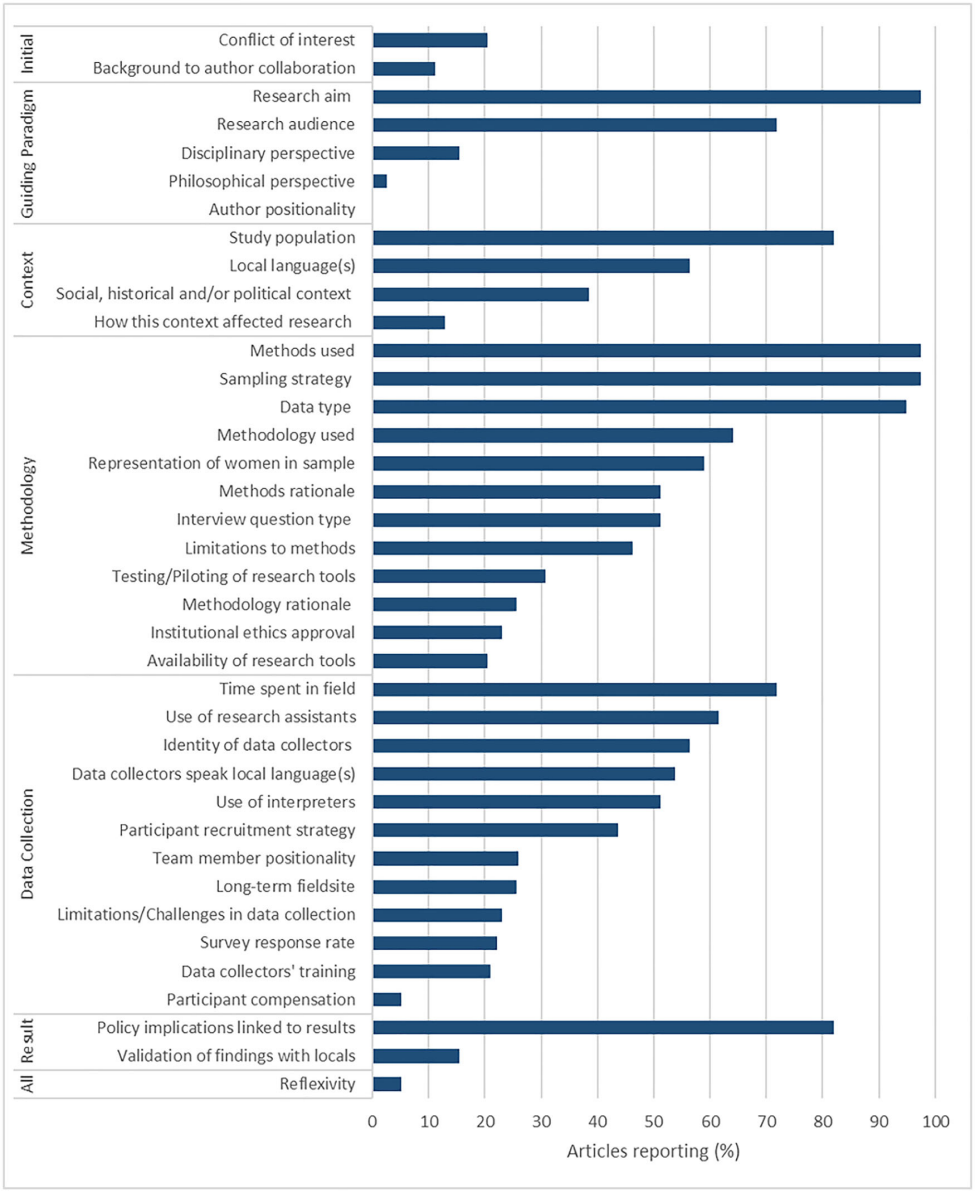
Percentage of articles in a literature review reporting on criteria applied to assess transparency in the research process (top to bottom, most to least commonly reported in each research stage; 39 papers reviewed; of these, 36 coauthored papers were assessed relative to the background of author collaboration; 27 papers reporting the use of survey methods were assessed relative to survey response rate; 38 papers for which the author was not the sole data collector were assessed relative to data collector training).

Of our selected transparency criteria (38), only 4 were consistently reported on across papers: research aim, methods used, sampling strategy, and data type (97% of 39 papers providing information in each case) (Figure 1). The percentage of papers reporting on the remaining 34 transparency criteria ranged from 0% to 82% (median = 28%, mean = 37%) (Figure 1). We considered these criteria within the categories of research stage, although some criteria (such as reporting on reflexivity) cut across the research process.

### Inception of research

All but 3 papers had multiple authors (range: 2–10 authors). The majority of affiliations (68.8% of the 157 affiliations across 148 authors) were to universities, 12.7% to research institutes, 10.2% to nongovernmental organizations (NGOs), and the remainder to consulting (3.2%), the public sector (3.2%), and other (1.9%). Twenty papers had affiliations across one or more sectors. Most first authors were affiliated with a university (33 of 39). Together, the reviewed papers encompassed fieldwork in 29 countries. Nearly half of papers reviewed (18) had no author affiliated with an institution located in the country where the research took place.

We identified 6 papers for which authors might have had a potential conflict of interest in results reported. Only 2 of these included a conflict‐of‐interest statement, of which only one disclosed information about the conflict (Sellers, 2019). In the other 4 papers, relationships between authors and the interventions being assessed were identifiable only through a careful reading of the article, acknowledgments, and listed affiliations (Green et al., 2011; Matiku et al., 2013; Owino et al., 2012; Sheppard et al., 2010).

Of the 36 papers with multiple authors, only 4 explained how authors were known to one another or came to do this research together (Figure 1). Although the aim of the research was almost universally reported, 11 studies were not explicit about who the research aimed to serve (Figure 1). Where the intended audience was mentioned, most authors targeted findings at policymakers, managers, or both (18 of 28 papers). Also mentioned were local residents or stakeholders (7), scholars or the literature (5), and donors (2).

### Guiding paradigm

No paper made explicit use of the terms *positionality* or *reflexivity* (Figure 1). Only 2 papers incorporated reflexive comments, for example, on the authors’ versus local community members’ subjectivities regarding social–ecological change (Andrachuk & Armitage, 2015). The same authors were the only ones to report on their philosophical perspective by acknowledging that their view on the existence of multiple realities had led to a research design that accessed different stakeholders’ perceptions of management interventions. This paper, and 5 others, also noted authors’ disciplinary (political ecology, anthropology, forestry) or transdisciplinary (socioecological systems) perspectives.

### Field site context

**FIGURE 2:**
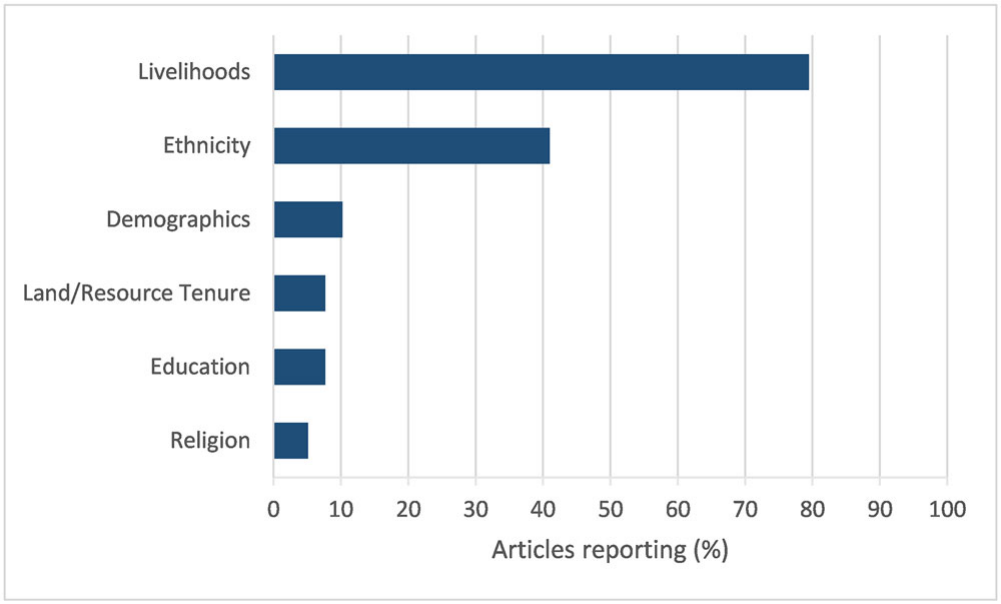
Percentage of articles in a literature review of transparency in conservation social science research in which authors reported on specific features of the study population (*n* = 39).

Nearly half of articles described the study population only in terms of livelihoods (28%) or not at all (18%); few other aspects were considered (Figure 2). Many articles (62%) provided no information on the wider historical or political context of the field setting (Figure 1). The remaining 15 articles provided some description, but this was mostly very limited, even where major events, such as civil war and colonization, were mentioned as affecting conservation. Five articles provided information on how the social, historical, and political context had affected the research process by, for example, describing how ongoing local resistance to conservation efforts led to initial distrust of the researchers and the steps taken to mitigate this problem (Mariki et al., 2015).

### Methodology

Most papers followed a mixed‐methods methodology (69%), rather than exclusively qualitative (18%) or quantitative (8%) (unclear in 2 of the 39 papers), but few authors justified their choice (Figure 1). Together, papers encompassed a wide range of methods, including various participatory methods (Appendix S7), but only half justified their methods (Figure 1). All papers reported using surveys, interviews, or both, but only half reported the question type, less than one‐third reported piloting or testing their research tools, and even fewer made their research tools available (Figure 1).

Information on whether women were represented in the study was lacking for 41% of papers (Figure 1). The majority (12 of 16 papers) also did not include information on whether other groups (e.g., stratified by wealth, marginalized status, occupation) were represented in their study.

Only about one quarter of articles reported on whether institutional ethical approval was obtained (Figure 1). Of these, 5 noted the use of prior informed consent (PIC) procedures, and 4 also stated that the research aims had been explained to participants. Two papers did not report any institutional ethics approval but did state using PIC and explaining research aims. We found no evidence as to whether researchers had additionally discussed their own values and motivations for the research with participants.

Nine papers described challenges to data collection, such as logistics (e.g., rainy season), difficulties with accessing certain groups, and reluctance of participants to engage with the topic or methodological tool. In one case, access to the field site was lost due to a deterioration in relationships with gatekeepers. None commented specifically on ethical challenges that might have arisen in the field.

### Data collection

Almost one third of articles did not specify the amount of time spent in the field (Figure 1). Ten articles noted that research was taking place at a long‐term field site and provided varying amounts of detail on who had spent time there and the start date of the relationship.

Clear identification (in the main body of the text or in an author contribution statement) of who undertook data collection was lacking in just over half of papers (Figure 1). Research assistants appeared to be widely relied on; 23 papers clearly reported on their use (or not) in the main body of the paper (Figure 1). An additional 4 articles referenced support from research assistants only in their “Acknowledgments.” Only 8 articles provided mention or information on training provided to data collectors, and only 7 provided an indication of the positionality of research assistants (Figure 1). In that regard, the most common descriptors were *local* and *experienced* or *expert* (3 mentions each), and only one paper reported on assistants’ gender (Appendix S8).

Linguistic diversity was a feature of our data set; 31 different languages were cited by the 22 papers providing this information (Appendix S9). However, it was difficult to establish whether research was carried out in the first language of participants. About half of papers specified that data collectors spoke the language spoken by residents of the field site (Figure 1). Where authors reported using interpreters (14 papers), the method of interpretation (e.g., on the spot, translation of recordings) was specified in only 6 cases.

Less than one half of papers reported on participant recruitment strategy, and only 2 made mention of participant compensation (Figure 1). How research tools were received by participants was rarely reported. For the 27 articles for which surveys were used, just 6 reported on the response rate.

### Findings

Every paper included a discussion of the policy implications of their research, and most linked these clearly to their findings (Figure 1). However, of the 7 papers with the stated aim of serving local communities, only 2 were among the 6 articles that reported validating their findings with local informants (Afriyie et al., 2021; Andrachuk & Armitage, 2015).

## DISCUSSION

Our findings illustrate consistent underreporting by authors of studies focused on protected areas and human well‐being in low‐ and middle‐income countries, which may reflect a broader problem with transparency in the field of conservation social science. Given this topic is particularly contested and authors may therefore be more careful to justify their methods and results, we have no reason to think that field research published on another topic in conservation social science would produce significantly different results. We did not attempt to analyze levels of transparency across different disciplines represented in our sample, in part because disciplinary perspective was not consistently reported (see below), but reporting that was not transparent appeared to occur across different types of studies. Overall, very few articles provided enough information for readers to gain insight into the researchers’ relationship to their team, to their participants, or to the wider society in which the study took place.

As set out in our positionality statement, our lived experience led us to expect a finding of inadequate levels of transparency in conservation social science reporting. As critical realists, we sought to interrogate this view through our literature review, but we also accept that our knowledge is only partial. Although none of the papers we surveyed made explicit mention of reflexivity, we are not suggesting that the authors were necessarily not reflexive in their research practices. Pienkowski et al. (2023) maintain that reflexivity is an abstract and enormous topic that conservationists may not name directly while still actively engaging in often informal, largely individual reflexive practices. Our point is that there is value in being more transparent about these reflexive processes, and the 2 are inherently linked.

We considered the implications of our key findings and devised recommendations. Table 1 outlines the key criteria often omitted from our sample, reformulated into guiding questions for researchers, reviewers, and editors to improve transparency in reporting while prompting reflection on their implications for research quality, equity, and collaboration.

**TABLE 1:** Questions for researchers, reviewers, and editors to consider to improve transparency and implications of the questions for reflexive practice, research quality (Q), equity (E), and collaboration (C).

Research stage	Question	Implication for reflexive practice and improved quality, equity, and collaboration
Inception	How did the research collaboration emerge and evolve, and what perspectives and strengths did the collaborators bring to the research?	Q: demonstrates disciplinary basis for collaborations and expertise in relation to the topic
		E: encourages consideration of equitable North–South collaboration and recognition of institutional imbalances
		C: models interdisciplinary collaboration for others, encourages reflection on disciplinary values and opportunities for improving disciplinary range within a team
	Do you, members of the research team, funders, partners, or supporting institutions have a personal interest in the research process or outcomes?	Q: disclosure can warn readers of potential bias (including unconscious) in findings
		E: encourages honest evaluations of researcher interests and power and how these have shaped the research and its potential impacts
Research values	How have your values, research philosophies, and disciplines shaped your research approach?	Q: demonstrates coherence between philosophy and methods and the ways in which the research is situated from a particular perspective (vital for confirmability)
		E: supports pathways to recognizing diverse and undervalued knowledge by acknowledging values and partiality of perspectives
		C: improves understanding and appreciation across subdisciplines including promoting reflexivity within multidisciplinary teams
	Who or what does the research aim to serve? What motivated you to conduct the research?	Q: allows readers to assess usefulness of the research in relation to its aim and the values and motivations of the researchers
		E: encourages explicit consideration of the implications of the research, which agendas it may support, and steps to link research to positive action and social justice
		C: encourages open discussions about motivations and alignment of goals in diverse teams
Methods	Why have the methods been chosen and what are their limitations in relation to the aim, context, and participants?	Q: supports accountability in research processes and provides information about the validity of the data
		E: encourages consideration of how methods are appropriate to the social context and gives recognition to diverse knowledge
		C: suggests opportunities to recruit different expertise and build multidisciplinary links for future research
	How did you decide who to include as participants? How were they recruited? Were different groups represented in the study, including the most marginalized?	Q: suggests whether results are representative of the community along relevant social strata
		E: works to widen participation and improve recognition of knowledge and experiences of diverse and marginalized groups
		C: reveals possible need for different expertise to access different participant groups and interpret data
	Did you pilot the use of your research tools? Did you need to adapt these during data collection? If so, why and how?	Q & C: reassures readers (including those from other disciplines) that tools were appropriate to research aims
		E: provides opportunities to tailor research tools to the local context and participant sensitivities
	Did you include standardized surveys, interview questions, or topic guides in supplementary materials?	Q: allows readers to understand data sources and issues that may affect credibility and transferability
		C: allows opportunities for other researchers to build on existing tools and protocols, advancing knowledge and avoiding duplication of work
	How does the social, political, and historical context affect the issue being examined and the research process?	Q: enables readers to discern transferability of results to other contexts and understand methodological decisions
		E: facilitates recognition of how the research and its impacts can work to rectify historical injustices and power imbalances
		C: leads to seeking out expertise for current and future research where gaps are recognized
Data collection	Who collected the data and how did their social position in relation to participants affect the research process?	Q: establishes researchers’ positionality helping the reader understand the ways in which the data are situated, e.g., ease of accessing certain people
		E: encourages reflection on how the research and its impacts may support or break down power imbalances and reveals potential for ethical harm
	Were research assistants involved and what is their experience, social position, and relation to the field site and participants?	Q: allows readers to assess whether issues such as gender, education, class, values, local experience, and training affected the data produced, e.g., by privileging certain voices or suppressing some opinions
		E: encourages consideration of recruitment and investment in skills training to promote diversity among field teams and more equitable relations with research participants
	Was the research carried out in the first language of all participants? If not, how was interpretation managed and validated?	Q: promotes awareness that language used denotes cultural knowledge and political status and may exclude some people from the research or result in misunderstandings
		E: comprehension and equal power are vital for free and informed consent
	How much time was spent at the field site (during the study and through longer term engagement)?	Q: suggests establishment of trust, relationships, and understanding of the context with likely improvements in the validity of research
		E: leads to improved opportunities for collaborative, efficacious research through the building of relationships over time
	Were free, prior, and informed consent processes followed? Were participants informed of researchers’ motivations and values?	Q: helps establish trusting relationships required for high‐quality research
		E: foundational for ethical research aligning with the rights to self‐determination of Indigenous peoples and local communities
	What was the response rate for surveys and what may have affected it? Were there issues accessing or engaging with participants?	Q: indicates credibility of the data by suggesting the level of comfort with the research and transferability in showing who did and did not participate
		E: encourages reflection on ways to improve relationships, ability to participate, and relevance of research for participants
	Were there any challenges in the fieldwork that affected the results? How did the methods work in practice in the context?	Q: helps readers assess validity and the context in which the data were produced, e.g., if accessing women on their own proved challenging
		C: suggests productive collaborations and where expertise may be needed, e.g., access to local knowledge
Analysis	Were results validated and fed back to communities involved?	Q: improves credibility of the data, interpretation, and likely impact of results
		E: redresses power imbalances as part of coproduced research and ensures research is meaningful and useful for communities
Discussion	How does the social, political, and historical context affect the feasibility and implications of any proposed recommendations from this research?	E: encourages reflection on how the findings are interpreted and promotes realistic proposals to deliver value to participants and maintain commitments to study communities

*Note*: Questions and implications drawn from Beck et al. (2021), Boyce et al. (2022), Brittain et al. (2020), Montana et al. (2020), Moon et al. (2016), and Montana et al. (2020) and authors' own work.

### Data quality

Although reporting on naturalistic criteria for assessing data quality has been recognized as important for qualitative social research in conservation (Moon et al., 2016), we suggest that such reporting applies to any social research, whether data are qualitative or quantitative, as part of reflexive practices. For example, researchers coming from a natural science background may use controls and randomization to discount confounding factors as a means of improving validity in social impact evaluations, but this does not preclude the need to ensure that surveys aimed at capturing human well‐being have been designed and carried out in a way that produces meaningful data in the naturalistic sense—such as by repeated engagement to build trust and understanding and by piloting survey questions to ensure they make sense to participants. If there is insufficient detail on these issues (as we found in many of the papers sampled), the reader cannot contextualize the results and must take it on trust that they are valid.

Details of what happened in the field were often scant. Relationships with research participants and engagement with the field site are particularly important in social data validity, but there was very little consideration of the former (beyond a few mentions of rapport building), and most papers did not report on the field experience. Although many studies used research assistants, their role in the research remained ambiguous and at times invisible, a recognized problem in social science (Turner, 2010). For example, Ulambayar et al. (2017) thank 23 assistants in their acknowledgments but make no mention of using assistants in the paper itself. The ability to assess quality is fundamentally undermined when readers cannot be sure who was spoken to, in what language, and with what level of trust or cultural and linguistic competency: the findings themselves become suspect.

Take for example the survey questionnaire, a widespread method in our sample and conservation social science generally. Response rates were almost never reported even though this metric can give insight into the trustworthiness of research findings. A low overall response rate to a survey might suggest that there are issues with the researcher's presence in the community or the nature of the questioning. It could indicate that only certain kinds of people are taking part. It might suggest that those who do participate are giving guarded answers, if there is a general unease around the exercise. The majority of papers did not publish survey or interview tools, making it impossible for the reader to even discern what kinds of questions had been asked and identify issues such as leading or ambiguous questions.

Challenges are almost inevitable during fieldwork in rural areas, and although authors reflected on limitations of methods, most did not provide information on how challenges may have affected the research outcomes. Rather than glossing over difficulties in recruitment, Corbera et al. (2020) usefully acknowledge that the overrepresentation of men and village officials means that their governance‐related findings should be treated with caution.

### Equity

Cross‐cultural research was common in our sample. Nearly half of papers reviewed had no author obviously affiliated with the country in which the research took place, which could suggest that aspects of parachute science were in play (Stefanoudis et al., 2021), although working on the basis of institutional address alone may obscure other links authors had to the research country and culture (Miller et al., 2023). Trisos et al. (2021) have called on researchers to reflect and report on their status as local or foreign, where foreignness can be measured across a wide set of social characteristics. Doing so, they argue, including through positionality statements (see below), would allow readers to better understand a paper's purpose, encourage researchers to reflect on the diversity and role of research collaborators, and contribute to decolonizing research practices. Because none of the papers reviewed provided positionality statements, only 4 discussed the background of authors’ collaborations, and few specified whether the research occurred at new or long‐term field sites, there was little alternative means besides institutional affiliation for readers to gauge the extent of authors’ connection to the field setting.

Several researchers implicitly recognized the importance of positionality by flagging the insider status of their research assistants as *local*. Yet, the term glosses over other important aspects of positionality that may affect relationships between the researchers and the researched, such as gender, education, class, religion, and caste. How might a so‐called local university graduate be perceived by a local farmer, for example? Would a female research assistant elicit different kinds of information from interlocutors than a male assistant? Identity may become less important if research assistants have local experience and long‐term connections to the field site, but in our sample, this was rarely reported. In avoiding descriptions of positionality, the way in which the research and knowledge produced are situated and partial may go unexamined. For example, where research assistants were identified not as locals but as professionals, the potential complications this might introduce were not discussed, as in a case using NGO staff to study communities involved with NGO activities. At the extreme end of the spectrum, there may be conflicts of interest that invalidate the findings. Potential conflict of interest issues among our sampled papers were few. However, worryingly, the majority of these not only failed to disclose any issues but also presented positive results of the interventions that the authors were involved in through working for either the implementing or funding organization.

The mention of local research assistants was also used to suggest to readers the language of data collection. In fact, establishing who exactly had collected the field data, and whether it was done in the primary language of informants, was astonishingly difficult. In multilingual contexts, where the elderly or more marginal groups in a community may not speak the dominant language, lack of clarity around the language used in data collection and the quality of interpretation was concerning. Language matters because it is intrinsically tied to cultural understanding and political positioning. Essential meaning can be lost in translation and affect data quality and equity (Broesch et al., 2023; Twyman et al., 1999). If participants do not fully understand what they are told about the research or feel at a disadvantage and obliged to participate in the study, this undermines the very principle of free and informed consent on which ethical research is based (Lewis, 2012).

Conservation science is an applied discipline, and all the papers, despite not always being explicit about the intended audience, discussed the implications for policy, practice, or both. Misrepresenting participants’ views—either through failing to access diverse and marginalized voices or failing to competently access and record them—reinforces power inequalities throughout the research process and in its impacts. We were surprised at the lack of information in many papers around participant representation in terms of gender. Similarly, there seemed to be little effort to stratify research samples by wealth, which suggests problematic assumptions that everyone in the study communities is equally poor. This is unlikely to be the case and is relevant to the impacts of protected areas, which tend to disproportionately negatively affect women, the poor, and the marginalized (Woodhouse et al., 2018). If these groups are not included or data are not disaggregated, these inequities will remain invisible, and opportunities to inform more socially just interventions will be missed. Coproducing research with communities is increasingly recognized as an important means of increasing quality and impact and redressing power imbalances. Only 6 of our sampled articles reported validating findings with the communities, a sample that did not completely overlap with papers stating an aim to serve local communities.

### Collaboration

Reflexivity—and transparent reporting of its outcomes—offers a basis for developing respectful collaboration across diverse disciplines and perspectives to achieve conservation aims in a socially just way (Boyce et al., 2022). Those few authors who did report on their disciplinary perspectives came from political ecology, social forestry, and environmental anthropology, which place greater emphasis on the social context of knowledge production. This finding perhaps signals a feeling of remaining outside of mainstream conservation social science, suggesting more progress is needed to widen the scope and inclusivity of the discipline (Bennett et al., 2017). The fact that only one paper reported on authors’ philosophical position could suggest that, across all disciplines, many conservation researchers are not reflecting on the norms and values that underlie their work and therefore did not see the need to report on them (Kareiva & Marvier, 2017; Pascual et al., 2021). More transparency around researchers’ theoretical orientations and the challenges (methodological, practical) that arose during fieldwork could identify opportunities to recruit different expertise to the research team and open up “productive pathways” for future research (Beck et al., 2021). For example, in our sample, an innovative Q‐sort methodology applied by Janssens et al. (2022) would likely have benefited from ethnographic expertise on the team to achieve more balanced representation.

Authors might not flag research preferences because they expect readers to deduce these from other clues, such as institutional affiliations, the choice of methodology, and the scope of the journal. Yet, given that many institutions, journals, and individuals now aim to be cross‐disciplinary in their approach, such heuristics can begin to break down. In reviewing papers, we were struck by the amount of mental energy often required to determine basic features of the research and contextualize research findings. Faced with this additional cognitive burden, people can make easy—and false—assumptions to fill gaps in understanding (Sweller, 1988). Reporting clearly on the principles, assumptions, and practicalities underlying research choices and findings could help build a more diverse readership—including practitioners, activists, and community representatives—and a stronger foundation for interdisciplinary collaboration.

### Recommendations

We recognize that there are different indicators of quality between positivist and interpretivist research, broadly corresponding to natural and social science approaches, respectively. Although these divisions do not run neatly along the quantitative–qualitative divide, that almost all papers in our sample incorporated qualitative data suggests a genuine concern with participants’ subjective experiences of conservation interventions, their interpretations, and the meanings they attached to outcomes. Nonetheless, postpositivism is the prevailing philosophical paradigm in conservation science (Moon et al., 2019); reflexivity is not well established, and publication norms remain tied to this perspective. Building transparency into published conservation papers will encourage, share learning about, and normalize reflexive practices.

We urge conservation scientists working with social data—qualitative or quantitative—to reach beyond the limits of their disciplinary norms and build opportunities for improving quality, equity, and collaboration into their research through reflexive practices, recognition of their roles and relationships within the research process, and transparency. Based on our findings and with this in mind, we recommend the following.

First, we advocate that journal editors, reviewers, and researchers shift their expectations of what is usefully reported in publications. In Table 1, we provide a set of questions based on issues that were poorly reported on in our review but that we consider important for providing evidence of and promoting research quality, equity, and collaboration. We aim for these to be useful prompts for conservation social scientists in initiating, carrying out, and reporting their research and for reviewers and editors in assessing transparency and reflexivity. Following Braun and Clarke's (2024) writing on qualitative standards in health research, we do not subscribe to the idea of universally applicable reporting checklists due to the diversity of philosophical positions, theoretical assumptions, and methods used in conservation social science. Rather, the questions and their applications should be seen as provocations to elicit reflexivity especially around guiding paradigms and data collection. Some questions (for instance, on research philosophy) will be widely applicable, and others less so. For example, piloting is not relevant to ethnographic studies, in which research themes emerge during fieldwork through an inductive approach. Validation by communities is a quality standard in postpositivist qualitative research but less important for interpretivist perspectives, in which meaning is seen as contextual. Nonetheless, feedback and discussion with communities will be important to build equitable research regardless of study type. Users will need to employ their disciplinary expertise to judge the applicability of these questions and how they approach them.

We understand that enhancing transparency may put pressure on publication word counts, but we suggest that a balance be struck whereby basic information (such as language and duration of fieldwork and recruitment and representation of participants) is required in the text with small adjustments to necessary word counts if required and more detailed reflexive discussions are included along with any survey or interview guides as supplementary materials.

Second, we encourage conservation social scientists to take the time to develop positionality statements and for editors to accommodate these for field‐based studies, review studies (such as ours), and other types of desk‐based research. Although all our sampled journals today require a declaration that research has been approved by an institutional ethics board, this does not go far enough in exploring ethical issues in a transparent way. As Ibbett and Brittain (2020) found in a review of ethics reporting in social science papers on wildlife hunting, specific safeguards for participants, including consent processes, are poorly reported. We further suggest that researchers reflect on power relations and ethical challenges that might have emerged during fieldwork. Similarly, author contribution statements, though valuable, do not necessarily make clear who was involved in data collection and cannot do so if these individuals are not among the authors (Sarna‐Wojcicki et al., 2017). Positionality statements, if well‐crafted, give valuable insight into the purpose of the article and the orientation and experience of the authors. One must, however, recognize the limitations of these statements and use the process of writing them as an opportunity to reflect and take active steps to decolonize research practices (Gani & Khan, 2024; Larocco et al., 2020). Statements should move beyond a simple accounting process listing identity characteristics and overlooking the real risks of exposing interlocutors to harm (Brittain et al., 2020). In Table 2, we provide guidance for conservation social scientists on how to prepare positionality statements.

**TABLE 2:** Suggested elements, reflexive prompts, and advice for preparing positionality statements.

What should a positionality statement do?	Questions to ask	Aspects to consider	Examples
Set out foundations for research collaboration	How did the collaboration emerge and evolve? What perspectives and strengths did collaborators bring?	Shared academic or personal histories, interests, and goals Complementarities in expertise or experience (e.g., disciplinary, ontological) Institutional imbalances	Archer et al., 2022; Boyce et al., 2022; Carothers et al., 2021; Trisos et al., 2021
Set out the intent of the project (or paper)	Who or what does the research aim to serve? What are its limitations?	Extent research aligns with intent Usefulness for intended audience Potential gaps or misrepresentation	
Set out perspectives of the researchers	How have your values, research philosophies, and disciplines shaped your research approach? How have your social characteristics shaped it?	Focus on most salient to situate readers quickly Expand consideration of positionality as needed in main text to clarify influence on access to field, researcher–researched relationships, and research decisions and outcomes (see list of attributes below)	
**Prompts: Which of my attributes may shape my research decisions and outcomes?**				
*Values*: beliefs, biases, preferences, philosophical perspectives (ontologies, epistemologies), political and ideological stances, and other *Social characteristics*: gender, ethnicity, age, ability or disability, religion, sexual orientation, economic status, class, caste, education, professional affiliations, nationality, immigration status, coloniality, rurality or urbanity, linguistic tradition, marital status, parental status, appearance, and others				
**Prompts: What might influence how others perceive me and my research?**				
*My own actions*: attire and behavior, experience, relationships and engagements, emotions, efforts to fit in and negotiate the field, etc. *Research team*: attributes and actions, relationships to one another and participants, etc. *Multiple positionalities of others*: their attributes, priorities, backgrounds, worldviews, etc. (Berger, 2015; Neely & Nguse, 2015; Sultana, 2007)				
**Suggestions**				
Write an initial positionality statement and return to it throughout the research (Beck et al., 2021). Consider how consistent your reality is with others: how do you know what you know (Milner, 2007)? No one has complete self‐knowledge and awareness. Be modest but radical in admitting absences and fallibilities (Rose, 1997) without becoming paralyzed by over‐essentializing (Idahosa & Bradbury, 2020) and guarding yourself against overexposure (Massoud, 2022). Recognize the limits of positionality statements and take steps to actively decolonize knowledge production (Gani & Khan, 2024; LaRocco et al., 2020).				

Third, we encourage conservationists to engage with appropriate tools for reflexivity and build them into regular practice for all team members. Recognizing that “many conservation biologists still lack the tools and support to transparently identify their underlying normative values and beliefs,” Boyce et al. (2022) call for a discipline‐wide conversation to develop and apply formal reflexivity methodologies—at the self, interpersonal, and collective levels. Our set of prompts and considerations (Table 1) can be purposefully integrated into discussions to shape research and its outputs, with particular relevance to field‐based social studies. Broader practical tools already exist, including Beck et al.’s (2021) tenets and questions focused around values, partnerships, histories, and impacts and Pienkowski et al.’s (2023) and Montana et al.’s (2020) prompts for conservation practitioners.

Finally, most researchers seemed to adopt the detached objectivity of a natural scientist in reporting on their research process, and many of the papers we reviewed were written in the passive voice (“Interviews were conducted…”). We recommend that authors make the simple but possibly radical shift to the first person in their writing to make visible their presence (and that of research assistants) and interactions in the social context and the implications and responsibilities that entails. Researchers are not automatons, and being more transparent about one's role in knowledge production matters for wider public understanding and trust (Thorpe, 2023).

We offer these suggestions in the spirit of making space for all researchers to reflect honestly on the challenges of doing conservation social science. We recognize that it is a learning process but that these are necessary conversations in the wider context of decolonizing conservation research and practice. Instead of adhering to the myth of value‐free knowledge in conservation social science, let us accept and be transparent about the inevitable messiness and limitations of research encounters and processes. Doing so will build a more authentic basis for ethical, collaborative, and effective conservation research and practice and provide better results for people and nature.

## Supporting information



Supporting information

## References

[cobi70003-bib-0001] Afriyie, J. O. , Asare, M. O. , Danquah, E. , & Pavla, H. (2021). Assessing the management effectiveness of three protected areas in Ghana. Conservation & Society, 19(1), 13–24.

[cobi70003-bib-0002] Andrachuk, M. , & Armitage, D. (2015). Understanding social‐ecological change and transformation through community perceptions of system identity. Ecology and Society, 20(4), Article 26.

[cobi70003-bib-0003] Archer, L. J. , Müller, H. S. , Jones, L. P. , Ma, H. D. , Gleave, R. A. , Cerqueira, A. D. , Hamilton, T. M. , & Shennan‐Farpón, Y. (2022). Towards fairer conservation: Perspectives and ideas from early‐career researchers. People and Nature, 4(3), 612–626.

[cobi70003-bib-0004] Attia, M. , & Edge, J. (2017). Be(com)ing a reflexive researcher: A developmental approach to research methodology. Open Review of Educational Research, 4(1), 33–45.

[cobi70003-bib-0005] Beck, J. M. , Elliott, K. C. , Booher, C. R. , Renn, K. A. , & Montgomery, R. A. (2021). The application of reflexivity for conservation science. Biological Conservation, 262, Article 109322.

[cobi70003-bib-0006] Bennett, N. J. , Roth, R. , Klain, S. C. , Chan, K. , Christie, P. , Clark, D. A. , Cullman, G. , Curran, D. , Durbin, T. J. , Epstein, G. , Greenberg, A. , Nelson, M. P. , Sandlos, J. , Stedman, R. , Teel, T. L. , Thomas, R. , Vérissimo, D. , & Wyborn, C. (2017). Conservation social science: Understanding and integrating human dimensions to improve conservation. Biological Conservation, 205, 93–108.

[cobi70003-bib-0007] Berger, R. (2015). Now I see it, now I don't: Researcher's position and reflexivity in qualitative research. Qualitative Research, 15(2), 219–234.

[cobi70003-bib-0008] Boyce, P. , Bhattacharyya, J. , & Linklater, W. (2022). The need for formal reflexivity in conservation science. Conservation Biology, 36(2), Article e13840. 34623701 10.1111/cobi.13840

[cobi70003-bib-0009] Bradley, S. , DeVito, N. , Lloyd, K. , Richards, G. , Rombey, T. , Wayant, C. , & Gill, P. (2020). Reducing bias and improving transparency in medical research: A critical overview of the problems, progress and suggested next steps. Journal of the Royal Society of Medicine, 113, 433–443. 33167771 10.1177/0141076820956799 PMC7673265

[cobi70003-bib-0010] Braun, V. , & Clarke, V. (2024). How do you solve a problem like COREQ? A critique of Consolidated Criteria for Reporting Qualitative Research. Methods in Psychology, 11, Article 100155. 10.1016/j.metip.2024.100155

[cobi70003-bib-0011] Brittain, S. , Ibbett, H. , de Lange, E. , Dorward, L. , Hoyte, S. , Marino, A. , Milner‐Gulland, E. J. , Newth, J. , Rakotonarivo, S. , Veríssimo, D. , & Lewis, J. (2020). Ethical considerations when conservation research involves people. Conservation Biology, 34(4), 925–933. 31953971 10.1111/cobi.13464

[cobi70003-bib-0012] Brockington, D. , & Wilkie, D. (2015). Protected areas and poverty. Philosophical Transactions of the Royal Society B: Biological Sciences, 370(1681), Article 20140271. 10.1098/rstb.2014.0271 PMC4614728 26460124

[cobi70003-bib-0013] Broesch, T. , Lew‐Levy, S. , Kärtner, J. , Kanngiesser, P. , & Kline, M. (2023). A roadmap to doing culturally grounded developmental science. Review of Philosophy and Psychology, 14(2), 587–609.

[cobi70003-bib-0014] Brosius, P. (2006). Common ground between anthropology and conservation biology. Conservation Biology, 20(3), 683–685. 16909554 10.1111/j.1523-1739.2006.00463.x

[cobi70003-bib-0015] Carothers, C. , Black, J. , Langdon, S. J. , Donkersloot, R. , Ringer, D. , Coleman, J. , Gavenus, E. R. , Justin, W. , Williams, M. , Christiansen, F. , Samuelson, J. , Stevens, C. , Woods, B. , Clark, S. J. , Clay, P. M. , Mack, L. , Raymond‐Yakoubian, J. , Sanders, A. A. , Stevens, B. L. , & Whiting, A. (2021). Indigenous peoples and salmon stewardship: A critical relationship. Ecology and Society, 26(1), Article 16. https://www.ecologyandsociety.org/vol26/iss1/art16/

[cobi70003-bib-0016] Chua, L. , Harrison, M. E. , Fair, H. , Milne, S. , Palmer, A. , Rubis, J. , Thung, P. , Wich, S. , Büscher, B. , Cheyne, S. M. , Puri, R. K. , Schreer, V. , Stepien, A. , & Meijaard, E. (2020). Conservation and the social sciences: Beyond critique and co‐optation. A case study from orangutan conservation. People and Nature, 2(1), 42–60.

[cobi70003-bib-0017] Collins, Y. A. , Maguire‐Rajpaul, V. A. , Krauss, J. E. , Asiyanbi, A. P. , Jiménez, A. , Mabele, M. B. , & Alexander‐Owen, M. (2021). Plotting the coloniality of conservation. Journal of Political Ecology, 28(1), 968–989.

[cobi70003-bib-0018] Corbera, E. , Martin, A. , Springate‐Baginski, O. , & Villaseñor, A. (2020). Sowing the seeds of sustainable rural livelihoods? An assessment of Participatory Forest Management through REDD plus in Tanzania. Land Use Policy, 97, Article 102962.

[cobi70003-bib-0019] Descola, P. (2005). On anthropological knowledge. Social Anthropology, 13(1), 65–73.

[cobi70003-bib-0020] Drury, R. , Homewood, K. , & Randall, S. (2011). Less is more: The potential of qualitative approaches in conservation research. Animal Conservation, 14(1), 18–24.

[cobi70003-bib-0021] Gani, J. , & Khan, R. (2024). Positionality statements as a function of coloniality: Interrogating reflexive methodologies. International Studies Quarterly, 68(2), Article sqae038.

[cobi70003-bib-0022] Gardner, C. J. (2021). Not teaching what we practice: Undergraduate conservation training at UK universities lacks interdisciplinarity. Environmental Conservation, 48(1), 65–70.

[cobi70003-bib-0023] Green, S. J. , White, A. T. , Christie, P. , Kilarski, S. , Meneses, A. B. T. , Samonte‐Tan, G. , Karrer, L. B. , Fox, H. , Campbell, S. , & Claussen, J. D. (2011). Emerging marine protected area networks in the coral triangle: Lessons and way forward. Conservation and Society, 9(3), 173–188.

[cobi70003-bib-0024] Guba, E. G. (1981). Criteria for assessing the trustworthiness of naturalistic inquiries. ECTJ ‐ Educational Communication and Technology Journal, 29(2), 75–91.

[cobi70003-bib-0025] Guillemin, M. , & Gillam, L. (2004). Ethics, reflexivity, and “Ethically important moments” in research. Qualitative Inquiry, 10(2), 261–280.

[cobi70003-bib-0026] Holmes, G. , Sandbrook, C. , & Fisher, J. A. (2016). Understanding conservationists' perspectives on the new‐conservation debate. Conservation Biology, 31(2), 353–363. 27558699 10.1111/cobi.12811 PMC6849763

[cobi70003-bib-0027] Ibbett, H. , & Brittain, S. (2020). Conservation publications and their provisions to protect research participants. Conservation Biology, 34, 80–92. 31016794 10.1111/cobi.13337 PMC7028057

[cobi70003-bib-0028] Idahosa, G. E. O. , & Bradbury, V. (2020). Challenging the way we know the world: Overcoming paralysis and utilising discomfort through critical reflexive thought. Acta Academica, 52(1), 31–53.

[cobi70003-bib-0029] Janssens, I. , de Bisthoven, L. J. , Rochette, A. J. , Kakai, R. G. , Akpona, J. D. T. , Dahdouh‐Guebas, F. , & Huge, J. (2022). Conservation conflict following a management shift in Pendjari National Park (Benin). Biological Conservation, 272, Article 109598.

[cobi70003-bib-0030] Joshi, M. , & Bhardwaj, P. (2018). Impact of data transparency: Scientific publications. Perspectives in Clinical Research, 9(1), 31–36. 29430415 10.4103/picr.PICR_104_17 PMC5799949

[cobi70003-bib-0031] Kareiva, P. M. , & Marvier, M. (2017). Uncomfortable questions and inconvenient data in conservation science. In P. M. Kareiva , M. Marvier , & B. R. Silliman (Eds.), Effective conservation science: Data not dogma (pp. 3–9). Oxford University Press.

[cobi70003-bib-0032] LaRocco, A. , Shinn, J. , & Madise, K. (2020). Reflections on positionalities in social science fieldwork in northern Botswana: A call for decolonizing research. Politics & Gender, 16, 845–873.

[cobi70003-bib-0033] Lewis, J. (2012). How to implement free, prior informed consent (FPIC). Participatory Learning and Action, 65, 175–178.

[cobi70003-bib-0034] Lincoln, Y. S. , & Guba, G. E. (1985). Naturalistic inquiry. Sage Publications.

[cobi70003-bib-0035] MacKenzie, C. A. (2016). Filtered meaning: Appreciating linguistic skill, social position and subjectivity of interpreters in cross‐language research. Qualitative Research, 16, 167–182.

[cobi70003-bib-0036] MacKenzie, J. M. (1988). The empire of nature: Hunting, conservation and British imperialism. Manchester University Press.

[cobi70003-bib-0037] Mariki, S. B. , Svarstad, H. , & Benjaminsen, T. A. (2015). Elephants over the cliff: Explaining wildlife killings in Tanzania. Land Use Policy, 44, 19–30.

[cobi70003-bib-0038] Marsden, E. (2020). Methodological transparency and its consequences for the quality and scope of research. In J. McKinley & H. Rose (Eds.), Routledge handbook of research methods in applied linguistics (1st ed., pp. 15–28). Routledge.

[cobi70003-bib-0039] Mascia, M. B. , Brosius, J. P. , Dobson, T. A. , Forbes, B. C. , Horowitz, L. , McKean, M. A. , & Turner, N. J. (2003). Conservation and the social sciences. Conservation Biology, 17(3), 649–650.

[cobi70003-bib-0040] Massoud, M. F. (2022). The price of positionality: Assessing the benefits and burdens of self‐identification in research methods. Journal of Law and Society, 49(1), S64–S86.

[cobi70003-bib-0041] Matiku, P. , Caleb, M. , & Callistus, O. (2013). The impact of participatory forest management on local community livelihoods in the Arabuko‐Sokoke Forest, Kenya. Conservation & Society, 11(2), 112–129.

[cobi70003-bib-0042] Miller, J. , White, T. B. , & Christie, A. P. (2023). Parachute conservation: Investigating trends in international research. Conservation Letters, 16(3), Article e12947. 10.1111/conl.12947

[cobi70003-bib-0043] Miller, T. R. , Minteer, B. , & Malan, L. C. (2011). The new conservation debate: The view from practical ethics. Biological Conservation, 144(3), 948–957.

[cobi70003-bib-0044] Milner, H. R. (2007). Race, culture and researcher positionality: Working through dangers seen, unseen and unforeseen. Educational Researcher, 36(7), 388–400.

[cobi70003-bib-0045] Montana, J. , Elliott, L. , Ryan, M. , & Wyborn, C. (2020). The need for improved reflexivity in conservation science. Environmental Conservation, 47(4), 217–219.

[cobi70003-bib-0046] Montana, J. , Sandbrook, C. , Robertson, E. , & Ryan, M. (2019). Revealing research preferences in conservation science. Oryx, 55(3), 404–411.

[cobi70003-bib-0047] Moon, K. , Blackman, D. A. , Adams, V. M. , Colvin, R. M. , Davila, F. , Evans, M. C. , Januchowski‐Hartley, S. R. , Bennett, N. J. , Dickinson, H. , Sandbrook, C. , Sherren, K. , St‐John, F. A. V. , van Kerkhoff, L. , & Wyborn, C. (2019). Expanding the role of social science in conservation through an engagement with philosophy, methodology, and methods. Methods in Ecology and Evolution, 10(3), 294–302.

[cobi70003-bib-0048] Moon, K. , Brewer, T. D. , Januchowski‐Hartley, S. R. , Adams, V. M. , & Blackman, D. A. (2016). A guideline to improve qualitative social science publishing in ecology and conservation journals. Ecology and Society, 21(3), Article 17.

[cobi70003-bib-0049] Nast, H. J. (1994). Women in the field: Critical feminist methodologies and theoretical perspectives. Professional Geographer, 46(1), 54–66.

[cobi70003-bib-0050] Neely, A. H. , & Nguse, T. (2015). Relationships and research methods: Entanglements, intra‐actions and diffraction. In T. Perreault , G. Bridge , & J. McCarthy (Eds.), The Routledge handbook of political ecology (pp. 140–149). Routledge.

[cobi70003-bib-0051] Owino, A. O. , Jillo, A. H. , & Kenana, M. L. (2012). Socio‐economics and wildlife conservation of a peri‐urban national park in central Kenya. Journal for Nature Conservation, 20(6), 384–392.

[cobi70003-bib-0052] Pascual, U. , Adams, W. M. , Díaz, S. , Lele, S. , Mace, G. M. , & Turnhout, E. (2021). Biodiversity and the challenge of pluralism. Nature Sustainability, 4(7), 567–572.

[cobi70003-bib-0053] Peterson, R. B. , Russell, D. , West, P. , & Brosius, J. P. (2010). Seeing (and doing) conservation through cultural lenses. Environmental Management, 45(1), 5–18. 18592304 10.1007/s00267-008-9135-1

[cobi70003-bib-0054] Pienkowski, T. , Kiik, L. , Catalano, A. , Hazenbosch, M. , Izquierdo‐Tort, S. , Khanyari, M. , Kutty, R. , Martins, C. , Nash, F. , Saif, O. , & Sandbrook, C. (2023). Recognizing reflexivity among conservation practitioners. Conservation Biology, 37(2), Article e14022. 36285608 10.1111/cobi.14022

[cobi70003-bib-0055] Powers, S. , & Hampton, S. (2019). Open science, reproducibility, and transparency in ecology. Ecological Applications, 29, Article e01822. 30362295 10.1002/eap.1822

[cobi70003-bib-0056] Redpath, S. M. , Young, J. , Evely, A. , Adams, W. M. , Sutherland, W. J. , Whitehouse, A. , Amar, A. , Lambert, R. A. , Linnell, J. D. , Watt, A. , & Gutíerrez, R. J. (2013). Understanding and managing conservation conflicts. Trends in Ecology & Evolution, 28(2), 100–109. 23040462 10.1016/j.tree.2012.08.021

[cobi70003-bib-0057] Rose, G. (1997). Situating knowledges: Positionality, reflexivities and other tactics. Progress in Human Geography, 21(3), 305–320.

[cobi70003-bib-0058] Sandbrook, C. (2017). Weak yet strong: The uneven power relations of conservation. Oryx, 51(3), 379–380.

[cobi70003-bib-0059] Sandbrook, C. , Fisher, J. A. , Holmes, G. , Luque‐Lora, R. , & Keane, A. (2019). The global conservation movement is diverse but not divided. Nature Sustainability, 2(4), 316–323.

[cobi70003-bib-0060] Sarna‐Wojcicki, D. , Perret, M. , Eitzel, M. V. , & Fortmann, L. (2017). Where are the missing coauthors? Authorship practices in participatory research. Rural Sociology, 82(4), 713–746.

[cobi70003-bib-0061] Sellers, S. (2019). Does doing more result in doing better? Exploring synergies in an integrated population, health and environment project in East Africa. Environmental Conservation, 46(1), 43–51. 30853746 10.1017/S037689291800022X PMC6400278

[cobi70003-bib-0062] Sheppard, D. J. , Moehrenschlager, A. , McPherson, J. M. , & Mason, J. J. (2010). Ten years of adaptive community‐governed conservation: Evaluating biodiversity protection and poverty alleviation in a West African hippopotamus reserve. Environmental Conservation, 37(3), 270–282.

[cobi70003-bib-0063] St John, F. A. V. , Keane, A. M. , Jones, J. P. G. , & Milner‐Gulland, E. J (2014). Robust study design is as important on the social as it is on the ecological side of applied ecological research. Journal of Applied Ecology, 51(6), 1479–1485.

[cobi70003-bib-0064] Stefanoudis, P. V. , Licuanan, W. Y. , Morrison, T. H. , Talma, S. , Veitayaki, J. , & Woodall, L. C. (2021). Turning the tide of parachute science. Current Biology, 31(4), R184–R185. 33621503 10.1016/j.cub.2021.01.029

[cobi70003-bib-0065] Stirling, A. , & Burgman, M. (2021). Strengthening conservation science as a crisis discipline by addressing challenges of precaution, privilege, and individualism. Conservation Biology, 35, 1738–1746. 34405462 10.1111/cobi.13809

[cobi70003-bib-0066] Sultana, F. (2007). Reflexivity, positionality and participatory ethics: Negotiating fieldwork dilemmas in international research. ACME: An International E‐Journal for Critical Geographies, 6(3), 374–385.

[cobi70003-bib-0067] Sweller, J. (1988). Cognitive load during problem solving: Effects on learning. Cognitive Science, 12, 257–285.

[cobi70003-bib-0068] Tallis, H. , & Lubchenco, J. (2014). A call for inclusive conservation. Nature, 515(7525), 27–28. 25373659 10.1038/515027a

[cobi70003-bib-0069] Thorpe, H. H. (2023). It matters who does science. Science, 380(6648), 873. 37262175 10.1126/science.adi9021

[cobi70003-bib-0070] Trisos, C. H. , Auerbach, J. , & Katti, M. (2021). Decoloniality and anti‐oppressive practices for a more ethical ecology. Nature Ecology & Evolution, 5(9), 1205–1212. 34031567 10.1038/s41559-021-01460-w

[cobi70003-bib-0071] Turner, S. (2010). Research Note: The silenced assistant. Reflections of invisible interpreters and research assistants. Asia Pacific Viewpoint, 51(2), 206–219.

[cobi70003-bib-0072] Tuval‐Mashiach, R. (2017). Raising the curtain: The importance of transparency in qualitative research. Qualitative Psychology, 4(2), 126–138.

[cobi70003-bib-0073] Twyman, C. , Morrison, J. , & Sporton, D. (1999). The final fifth: Autobiography, reflexivity and interpretation in cross‐cultural research. Area, 31(4), 313–325.

[cobi70003-bib-0074] Ulambayar, T. , Fernández‐Giménez, M. E. , Baival, B. , & Batjav, B. (2017). Social outcomes of community‐based rangeland management in Mongolian steppe ecosystems. Conservation Letters, 10(3), 317–327.

[cobi70003-bib-0075] Woodhouse, E. , Bedelian, C. , Barnes, P. , Cruz‐Garcia, G. S. , Dawson, N. , Gross‐Camp, N. , Homewood, K. , Jones, J. P. G. , Martin, A. , Morgera, E. , & Schreckenberg, K. (2022). Rethinking entrenched narratives about protected areas and human wellbeing in the Global South. UCL Open Environment, 4, Article e050. 10.14324/111.444/ucloe.000050 37228477 PMC10208335

[cobi70003-bib-0076] Woodhouse, E. , Bedelian, C. , Dawson, N. , & Barnes, P. (2018). Social impacts of protected areas: Exploring evidence of trade‐offs and synergies. In G. Mace , K. Schreckenberg , & M. Poudyal (Eds.), Ecosystem services and poverty alleviation: Trade‐offs and governance (pp. 305–316). Routledge.

[cobi70003-bib-0077] Young, J. C. , Rose, D. C. , Mumby, H. S. , Benitez‐Capistros, F. , Derrick, C. J. , Finch, T. , Garcia, C. , Home, C. , Marwaha, E. , Morgans, C. , Parkinson, S. , Shah, J. , Wilson, K. A. , & Mukherjee, N. (2018). A methodological guide to using and reporting on interviews in conservation science research. Methods in Ecology and Evolution, 9(1), 10–19.

