# Supplementary material for: Improving transparency in conservation social science research to enhance quality, equity, and collaboration
Source: Conserv Biol. 2025 Apr 1;39(2):e70003. doi: 10.1111/cobi.70003 (PMC11959340; doi:10.1111/cobi.70003)
Supplement: Supplementary file 1 — Supporting information [file COBI-39-e70003-s001.docx]

**Supporting Information**

Appendix S1.

Literature search strategy - In addition to papers drawn from an existing database (described in Woodhouse et al. 2022) we conducted our own literature search in the Web of Science (WoS) database for papers published from 2018-2022 inclusive (Table S1). We used identical keywords to those given in Woodhouse et al. (2022).

Some journals, for some years (2018 – 2020), were initially searched manually, by one author (MAM) reading through online Tables of Content for all issues and recording candidate papers in a notebook (Table S1). We later repeated the search in WoS for each of these journals to check for consistency in generating the pool of candidate papers. We found that (1) all candidate papers identified manually also appeared in the WoS results; (2) only one paper was picked up in the WoS search that I had not found manually and (3) we had recorded 2 papers that did not appear in the WoS results (but these were not randomly selected for inclusion in our analysis).

Table S1. Sources used to identify candidate papers for our sampling pool.

| Journal | Existing Database  (2010-2017) | Web of Science  (2018-2022 |
| --- | --- | --- |
| Ambio | x | x |
| Biodiversity & Conservation | x | x |
| Biological Conservation | x | x |
| Conservation Biology | x | x |
| Conservation Letters | x | x |
| Conservation & Society | x | x |
| Ecology & Society | x | x^1^ |
| Environmental Conservation | x | x^1^ |
| Journal for Nature Conservation | x | x^1^ |
| Journal of Environmental Management | x | x^1^ |
| Land Use Policy | x | x^1^ |
| People and Nature | n/a | x (2021 only) |

^1^ These journals were initially searched manually by one author (MAM) reading through the online table of contents for all issues published from 2018 – 2020 inclusive. This was for reasons of convenience as I could browse these easily on my smartphone when away from my desk.

Appendix S2.


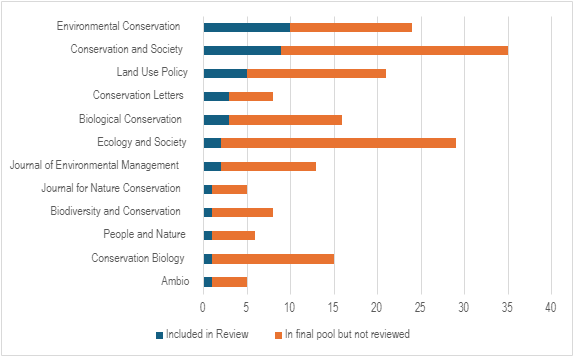


Figure S2. Total number of articles that were in our final pool (N = 185), showing the total number included in the review (N = 39), per journal.

Income-level of countries in which fieldwork was conducted in our sample of reviewed papers:

**Low-Income Countries**: Benin, Central African Republic, Democratic Republic of Congo, Ethiopia, Guinea-Bissau, Madagascar, Mozambique, Nepal, Rwanda, Sierra Leone, Tanzania, Uganda, and Zambia.

**Lower Middle-Income Countries**: Belize, Bhutan, Bolivia, Ghana, India, Indonesia, Kenya, Kiribati, Lao PDR, Mongolia, Papua New Guinea, Philippines, Republic of Congo, and Vietnam.

**Upper Middle-Income Countries:** Ecuador and Peru. NB. These are included in the sample because one paper reviewed (Heyman & Stronza 2011) had fieldsites in Belize, Bolivia, Ecuador and Peru. Belize was lower-middle income in 2011, moving to the upper-middle income category in 2012; Bolivia remains lower middle income.

Our starting database used the World Bank’s income categories from March 2017 to categorise countries as low or lower-middle income, with the list reproduced in Woodhouse et al. (2022). As countries can be re-categorized each year, we checked if papers randomly selected for inclusion in our review and published after 2018 still met the low/lower-middle income inclusion criteria by checking the online World Bank Database.

**Appendix S3.**

Articles included in the review sample:

Afriyie, J. O., Asare, M. O., Danquah, E. and Pavla, H. (2021) 'Assessing the Management Effectiveness of Three Protected Areas in Ghana, *Conservation & Society,* 19(1).

Ahmed, A. and Gasparatos, A. (2020) 'Reconfiguration of land politics in community resource management areas in Ghana: Insights from the Avu Lagoon CREMA', *Land Use Policy,* 97.

Andrachuk, M. and Armitage, D. (2015) 'Understanding social-ecological change and transformation through community perceptions of system identity', *Ecology and Society,* 20(4).

Aymoz, B. G. P., Randrianjafy, V. R., Randrianjafy, Z. J. N. and Khasa, D. P. (2013) 'Community management of natural resources: A case study from Ankarafantsika National Park, Madagascar', *Ambio,* 42(6), pp. 767-775.

Bidaud, C., Schreckenberg, K., Rabeharison, M., Ranjatson, P., Gibbons, J. and Jones, J. P. G. (2017) 'The Sweet and the Bitter: Intertwined Positive and Negative Social Impacts of a Biodiversity Offset', *Conservation and Society,* 15(1), pp. 1-13.

Cinner, J. and Huchery, C. (2014) 'A Comparison of Social Outcomes Associated with Different Fisheries Co-Management Institutions', *Conservation Letters,* 7(3), pp. 224-232.

Corbera, E., Martin, A., Springate-Baginski, O. and Villaseñor, A. (2020) 'Sowing the seeds of sustainable rural livelihoods? An assessment of Participatory Forest Management through REDD plus in Tanzania', *Land Use Policy,* 97.

Dawson, N., Martin, A. and Danielsen, F. (2018) 'Assessing Equity in Protected Area Governance: Approaches to Promote Just and Effective Conservation', *Conservation Letters,* 11(2).

Diedrich, A., Stoeckl, N., Gurney, G. G., Esparon, M. and Pollnac, R. (2017) 'Social capital as a key determinant of perceived benefits of community-based marine protected areas', *Conservation Biology,* 31(2), pp. 311-321.

Green, S. J., White, A. T., Christie, P., Kilarski, S., Meneses, A. B. T., Samonte-Tan, G., Karrer, L. B., Fox, H., Campbell, S. and Claussen, J. D. (2011) 'Emerging marine protected area networks in the coral triangle: Lessons and way forward', *Conservation and Society,* 9(3  ), pp. 173-188.

Harihar, A., Ghosh-Harihar, M. and MacMillan, D. C. (2014) 'Human resettlement and tiger conservation - Socio-economic assessment of pastoralists reveals a rare conservation opportunity in a human-dominated landscape', *Biological Conservation,* 169, pp. 167-175.

Hartter, J., Solomon, J., Ryan, S. J., Jacobson, S. K. and Goldman, A. (2014) 'Contrasting perceptions of ecosystem services of an African forest park', *Environmental Conservation,* 41(4), pp. 330-340.

Heyman, W. D. and Stronza, A. (2011) 'South-South Exchanges Enhance Resource Management and Biodiversity Conservation at Various Scales', *Conservation and Society,* 9(2), pp. 146-158

Hoshino, E., van Putten, I., Girsang, W., Resosudarmo, B. P. and Yamazaki, S. (2016) 'A Bayesian belief network model for community-based coastal resource management in the Kei Islands, Indonesia', *Ecology and Society,* 21(2).

Janssens, I., de Bisthoven, L. J., Rochette, A. J., Kakai, R. G., Akpona, J. D. T., Dahdouh-Guebas, F. and Huge, J. (2022) 'Conservation conflict following a management shift in Pendjari National Park (Benin)', *Biological Conservation,* 272.

Johnson, M. F., Karanth, K. K. and Weinthal, E. (2018) 'Compensation as a Policy for Mitigating Human-wildlife Conflict Around Four Protected Areas in Rajasthan, India', *Conservation & Society,* 16(3), pp. 305-319.

Karki, S. T. (2013) 'Do protected areas and conservation incentives contribute to sustainable livelihoods? A case study of Bardia National Park, Nepal', *Journal of Environmental Management,* 128, pp. 988-999.

Larson, L. R., Conway, A. L., Hernandez, S. M. and Carroll, J. P. (2016) 'Human-wildlife Conflict, Conservation Attitudes, and a Potential Role for Citizen Science in Sierra Leone, Africa', *Conservation & Society,* 14(3), pp. 205-217.

Llopis, J. C., Diebold, C. L., Schneider, F., Harimalala, P. C., Andriamihaja, O. R., Messerli, P. and Zaehringer, J. G. (2023) 'Mixed impacts of protected areas and a cash crop boom on human well-being in North-Eastern Madagascar', *People and Nature,* 5(6), pp. 1786-1803.

Mariki, S. B., Svarstad, H. and Benjaminsen, T. A. (2015) 'Elephants over the Cliff: Explaining Wildlife Killings in Tanzania', *Land Use Policy,* 44, pp. 19-30.

Matiku, P., Caleb, M. and Callistus, O. (2013) 'The Impact of Participatory Forest Management on Local Community Livelihoods in the Arabuko-Sokoke Forest, Kenya', *Conservation & Society,* 11(2), pp. 112-129.

Mavah, G. A., Funk, S. M., Child, B., Swisher, M. E., Nasi, R. and Fa, J. E. (2018) 'Food and livelihoods in park-adjacent communities: The case of the Odzala Kokoua National Park', *Biological Conservation,* 222, pp. 44-51.

Narayan, T., Sherub, S. and Root-Bernstein, M. (2022) 'A culturally appropriate redesign of the roles of protected areas and community conservation: understanding the features of the Wangchuck Centennial National Park, Bhutan', *Biodiversity and Conservation*.

Nkhata, B. A. and Breen, C. M. (2010) 'Performance of community-based natural resource governance for the Kafue Flats (Zambia)', *Environmental Conservation,* 37(3), pp. 296-302.

Owino, A. O., Jillo, A. H. and Kenana, M. L. (2012) 'Socio-economics and wildlife conservation of a peri-urban national park in central Kenya', *Journal for Nature Conservation,* 20(6), pp. 384-392.

Parker, P., Thapa, B. and Jacob, A. (2015) 'Decentralizing conservation and diversifying livelihoods within Kanchenjunga Conservation Area, Nepal', *Journal of Environmental Management,* 164, pp. 96-103.

Pereira, J., Rosalino, L. M., Mucova, S., Massangue, Y., Abdulrazak, M., Vahossa, S., Selemane, M., Fonseca, C. and Santos, M. J. (2021) 'Livelihood vulnerability increases human-wildlife interactions', *Environmental Conservation,* 48(4), pp. 301-309.

Randriamady, H. J., Park, S., Andrianarimanana, D., Berobia, A. and Golden, C. D. (2021) 'The effect of conservation policies on wildlife hunting and consumption in north-eastern Madagascar', *Environmental Conservation,* 48(3), pp. 225-232.

Robinson, C. A. J., Daspit, L. L. and Remis, M. J. (2011) 'Multi-faceted approaches to understanding changes in wildlife and livelihoods in a protected area: a conservation case study from the Central African Republic', *Environmental Conservation,* 38(2), pp. 247-255.

Scheba, A. and Rakotonarivo, O. S. (2016) 'Territorialising REDD plus : Conflicts over market-based forest conservation in Lindi, Tanzania', *Land Use Policy,* 57, pp. 625-637.

Sellers, S. (2019) 'Does Doing More Result in Doing Better? Exploring Synergies in an Integrated Population, Health and Environment Project in East Africa', *Environmental Conservation,* 46(1), pp. 43-51.

Sheppard, D. J., Moehrenschlager, A., McPherson, J. M. and Mason, J. J. (2010) 'Ten years of adaptive community-governed conservation: evaluating biodiversity protection and poverty alleviation in a West African hippopotamus reserve', *Environmental Conservation,* 37(3), pp. 270-282.

Solomon, J., Jacobson, S. K. and Liu, I. (2012) 'Fishing for a solution: can collaborative resource management reduce poverty and support conservation?', *Environmental Conservation,* 39(1), pp. 51-61.

Temudo, M. P. (2012) 'The white men bought the forests: Conservation and contestation in Guinea-Bissau, Western Africa', *Conservation and Society,* 10(4), pp. 354-366

Teuea, T. and Nakamura, N. (2020) 'Motivations to Support Marine Conservation Projects in North Tarawa, Kiribati', *Conservation & Society,* 18(2), pp. 161-171.

Tolbert, S., Makambo, W., Asuma, S., Musema, A. and Mugabukomeye, B. (2019) 'The Perceived Benefits of Protected Areas in the Virunga-bwindi Massif', *Environmental Conservation,* 46(1), pp. 76-83.

Triguero-Mas, M., Olomí-Solà, M., Jha, N., Zorondo-Rodríguez, F. and Reyes-García, V. (2010) 'Urban and rural perceptions of protected areas: a case study in Dandeli Wildlife Sanctuary, Western Ghats, India', *Environmental Conservation,* 36(3), pp. 208-217.

Ulambayar, T., Fernández-Giménez, M. E., Baival, B. and Batjav, B. (2017) 'Social Outcomes of Community-based Rangeland Management in Mongolian Steppe Ecosystems', *Conservation Letters,* 10(3), pp. 317-327.

Wondirad, A. and Ewnetu, B. (2019) 'Community participation in tourism development as a tool to foster sustainable land and resource use practices in a national park milieu', *Land Use Policy,* 88.

**Appendix S4.**

Table S4. Criteria used in this study for evaluating transparency in reporting results of research on the social impacts of protected areas in conservation journals, with reference to supporting literature.

| **Research Stage** | **Criteria**  ***(Information provided - Yes/No)*** | **Justification and main source** |
| --- | --- | --- |
| *Inception* | Background to author collaboration | Can mitigate unequal relations associated with parachute science (Pascual et al. 2021, Trisos et al. 2021,) |
|  | Conflicts of interest | Enables disclosure of potential sources of bias, power and privilege (Hardwicke et al. 2020, Burgman et al. 2023) |
| *Research Values* | Author’s positionality | Authors’ characteristics influence all stages of the research process (Guillemin & Gillam 2004; Moon et al. 2016; Trisos et al. 2021) (S6 – SRQR) |
|  | Ontology, epistemology, philosophical perspective | Critical for readers to assess design and interpretation, and for respectful engagement with others (Moon et al. 2016, Boyce et al. 2022, Burgman et al. 2023) (S5 – SRQR) |
|  | Motivation and audience | Enables assessment of credibility, confirmability and basis for collaboration (Moon et al. 2016, Kareiva & Marvier 2017) (S4- SRQR) |
|  | Context | Features of setting and population needed to understand methodological decisions, how these might have affected findings, and their potential relevance (Miller et al. 2023) (S7 – SRQR) |
| *Methods* | Methodology and methods, with justification | Allows readers to consider whether methodological decisions and assumptions are appropriate and suitable for their own research (Moon et al. 2016, Young et al. 2018) (S5, S10 – SRQR) |
|  | Limitations to methods | As above. |
|  | Sampling strategy* | As a key component of methodological description (Moon et al. 2016, Young et al. 2018) (S8 – SRQR) |
|  | Sample representativeness* | Generalisability of conclusions depends on representativeness of group studied (Creswell 1994, Moon et al. 2016, Young et al. 2018) (S12 – SRQR) |
| **Research Stage** | **Criteria**  **(Information provided - Yes/No)** | **Justification and main source** |
|  | Research tools tested or piloted* | Testing and refining research tools should enhance data quality. (Young et al. 2018, Hardwicke et al. 2020) (S11 – SRQR) |
|  | Research tools available (incl. as supplementary material)* | Provide detailed evidence of how data were obtained (Hardwicke et al. 2020). |
| *Ethics* | Ethical issues and clearance | Documentation of approval by an appropriate ethics review board, or justification if otherwise, as an indication of ethical engagement (Ibbett & Brittain 2019; Brittain et al. 2020) (S9 – SRQR) |
| *Data collection* | Use of data collectors and/or research assistants | As a key component of methodological description (Newing 2011, Ch.11) and ethical obligation (Ibbett & Brittain 2019). |
|  | Positionality of research team members | The positionality of people involved in data collection (not only authors) will affect information collected (Turner 2010, Middleton & Cons 2014, Neely & Nguse 2015). (S6 – SRQR) |
|  | Training provided* | As a key component of methodological description (Newing 2011, Ch.11) |
|  | Language and interpretation | Inter-cultural communication complicates the interpretation and representation of knowledge (Twyman et al. 1999, Broesch et al. 2022) . |
|  | Time spent in field | Longer engagement equated to deeper relationships and better contextual knowledge and insights (Guba 1981, Bernard 2017, Ch.12) (S10 – SRQR) |
|  | Participant recruitment, compensation and response rate* | As an indication of ethical practice and to assess potential bias (Lewis 2012, Moon et al. 2016) |
|  | Limitations/Challenges with data collection | Allows assessment of bias and allows future research to mitigate issues identified (Young et al. 2018); may highlight ethical harm (Ibbett & Brittain 2019) (S19 – SRQR) |
| **Research Stage** | **Criteria**  **(Information provided - Yes/No)** | **Justification and main source** |
|  |  |  |
| *Analysis* | Validation of findings or involvement by local informants | As an indicator of data quality (Moon et al. 2016) (S15 – SRQR) |
| *Outcomes* | Empirical links to policy/practice recommendations | Evidence presented to support any recommendations to guard against stereotypical/ value-driven narratives (Beck et al. 2021) (S17 – SRQR) |
| *Reflexivity* | Reflexivity | Reflexivity and transparency are linked in supporting thoughtful decision-making around research, equity and collaboration (Moon et al. 2016, Stirling & Burgman 2020, Montana et al. 2020, Beck et al. 2021, Boyce et al. 2022, Pienkowski et al. 2022) (S6 – SRQR) |

**Criterion not relevant to all publications/methodologies (e.g., ethnography)*

*Notes on Table:*  For a fuller consideration of the implications for research quality, equity and collaboration associated with transparent reporting on these criteria, see Table 1. Where a criterion aligns with specific Standards for Reporting Qualitative Research (SRQR) (O’Brien et al. 2014) these are indicated in brackets. Criteria are arranged roughly by stages in the research process, though some cut across multiple stages.

**Additional references for Table if not already cited in main text:**

Bernard, H. R. (2017) *Research methods in anthropology: qualitative and quantitative approaches, 6th edition*. 6th ed. Rowman & Littlefield Publishers.

Creswell, J.W. (1994). *Research design: Qualitative and quantitative approaches*. Thousand Oaks: Sage Publications.

Hardwicke, T. E., Wallach, J.D., Kidwell, M.C., Bendixen, T., Cruwell, S. and Ioannidis, J.P.A. (2020) An empirical assessment of transparency and reproducibility-related research practices in the social sciences (2014–2017). *Royal Society Open Science* 7(2): 190806

Ibbett H and Brittain, S. (2019). Conservation publications and their provisions to protect research participants. *Conservation Biology* 34: 80–92.

Middleton, T. & Cons, J. (2014) Coming to terms: Reinserting research assistants into ethnography’s past and present. *Ethnography* 15 (3), 279–290.

Newing, H. (2011) *Conducting Research in Conservation: Social Science Methods and Practice*. 1st edition. Oxford: Routledge.

O’Brien, B. C., Harris, I.B, Beckman, T.J., Reed, D.A. and Cook, D.A. (2014) Standards for Reporting Qualitative Research: A Synthesis of Recommendations. *Academic medicine* 89 (9): 1245–1251.

Appendix S5. Codebook

| **Elements** | **Transparency Criteria?** | **Variable in dataset** | **How coded** |
| --- | --- | --- | --- |
| *Bibliographic & Background info* |  | Reviewer | MAM/EW |
|  |  | First Author Name | Text |
|  |  | Year of publication | Year |
|  |  | Journal | Text |
|  |  | Title of Paper | Text |
|  |  | Number of authors | Count |
|  |  | Country/ies of field work | Text |
|  | Y | Languages used in research stated | Y/N; if Y, what language(s) |
|  |  | Affiliation type Author 1 (Author 2… etc) | University, Public sector, Research Institute, Consultant, NGO, Industry, Unclear (can select more than one) |
|  |  | Cross-affiliation present (i.e., if more than two affiliation types represented on authorial team) | Y/N |
| *Inception* |  | Any indication of potential conflict of interest (whether noted by authors or not)? | Y/N; If Y add note |
|  | Y | Conflict of interest statement included | Y/N; If N, any conflict stated in text? If Y, add note |
|  |  | Number of authors affiliated to an organisation based in the country where research took place | Count |
|  | Y | Background to the author collaboration provided | Y/N (n/a for single author papers) |
| *Research Values* | Y | Positionality of authors explored | Y/N; If yes add notes on dimensions (as above) |
|  | Y | Philosophical perspective (ontology, epistemology) specified | Y/N; If Y, add notes |
|  | Y | Disciplinary perspective specified | Y/N; If Y, add notes |
|  | Y | Motivation for the research explained | Y/N |
|  | Y | Who the audience aims to serve specified | Y/N; If Y, add notes |
| *Wider context* | Y | Relevant aspects of the study population described | Y/N; If yes, add notes (e.g., Livelihoods, Religion, Ethnicity, etc.) |
|  | Y | Social, historical and political context described | Y/N |
| **Elements** | **Transparency Criteria?** | **Variable in dataset** | **How coded** |
| *Methods* | Y | Methodology specified (e.g., experimental, statistical, ethnography, action research, grounded theory, case study, etc) | Y/N; If Y, add notes |
|  | Y | Methodology justified | Y/N |
|  | Y | Methods specified (e.g., Survey, Interview, Focus Group, Participant observation, workshop, secondary data, Other) | Y/N; If other, specify |
|  | Y | Methods justified | Y/N; if Y add note |
|  | Y | Limitations to methods noted | Y/N |
|  | Y | Sampling strategy stated | Y/N; if Y add note |
|  | Y | Whether women represented in sample specified | Y/N |
|  | Y | Whether different wealth groups represented in sample specified | Y/N |
|  | Y | Whether marginalised groups represented in sample specified | Y/N (e.g., by livelihood, location, ethnicity, etc.) |
|  | Y | Whether other groups (not necessarily marginalised) represented in sample | Y/N; If yes, describe |
|  | Y | Data type stated | Y/N; if Y note qualitative, quantitative, mixed |
|  | Y | Interview question type stated in paper | Y/N (e.g., open, closed, mixed) |
|  | Y | Research tools tested or piloted stated | Y/N |
|  | Y | Interview guides/questionnaires provided | Y/N (as appendices, supplementary material) |
| *Ethics* | Y | Whether institutional ethics approval obtained specified (in main body or statement) | Y/N |
|  |  | Use of FPIC process stated | Y/N |
|  |  | Whether participants told research aims stated | Y/N; if Y add note |
| *Data collection* | Y | Data collectors specified clearly in main body of paper | Y/N |
|  |  | Whether research assistants used (as determined by reviewers from main body text and acknowledgements) | Y/N |
|  | Y | Whether research assistants used specified in main body of paper (i.e., not authors on paper) | Y/N |
|  | Y | Data collectors trained in relevant methods specified | Y/N |
| **Elements** | **Transparency Criteria?** | **Variable in dataset** | **How coded** |
|  | Y | Whether data collectors speak the language of field research stated | Y/N |
|  | Y | Whether research carried out in respondents first language stated | Y (in all cases)/ Y (in some cases)/N |
|  | Y | Whether Interpreters used stated | Y (always)/ Y (in some cases)/ N/no information; if yes, note method of translation or state ‘not specified’ |
|  | Y | Length of time spent in the field stated | Y/N; if Y note length of time |
|  | Y | Whether it’s a long term field site stated | Y/N; If yes, provide length of relationship with field site (in years) |
|  | Y | Participant recruitment described | Y/N |
|  | Y | Compensation for participation specified | Y/N |
|  | Y | Response rate provided (for surveys only) | Y/N |
|  | Y | Challenges in the field/ limitations related to data collection discussed | Y/N; If yes, add notes |
| *Analysis* | Y | Validation of analysis or involvement by local informants | Y/N |
| *Outcomes* |  | Policy/practice implications discussed | Y/N |
|  | Y | If yes, policy/practice implications link clearly to the research results | Y/N; if No, add notes |
| *Reflexivity* | Y | Specific mention of reflexivity in the paper | Y/N; If Y, add note |

Notes on codebook: Yes entered as 1 and No as 0 on datasheet. We did not address all transparency criteria listed here in our analysis, as some were difficult to score accurately. For example, authors might not specify whether all participants spoke the data collectors (or interpreters’) language as their own first language.

**Appendix S6. Trend analysis**


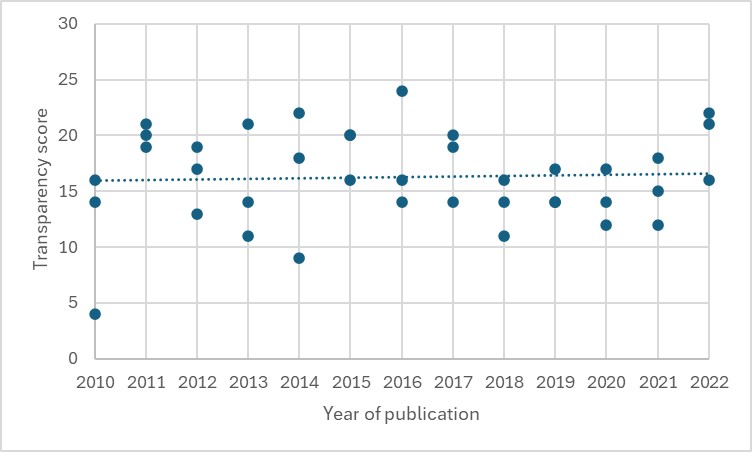


Figure S6. Trend analysis of transparency scores of sampled papers

Transparency score was calculated by totalling the criteria that were reported on in each paper. A linear trendline has been fitted to the data.

**Appendix S7.**

Table S7. Number of times various methods were reported as used in reviewed papers (N=39)

| Method | Frequency |
| --- | --- |
| Survey | 27 |
| Interview | 25 |
| Focus group | 12 |
| Life history interview | 1 |
| Workshop | 3 |
| Participant observation | 5 |
| Participatory exercises (free listing, timelines, card sorts, wealth ranking, wellbeing dialogues) | 4 |
| Participatory mapping | 1 |
| Q-sort | 1 |
| Transect walks | 1 |
| Follow-up field visits | 1 |
| Impressions of the researchers (autoethnography) | 1 |
| Ecological line transects | 1 |
| Secondary data (Review of program-related documents; Fiscal and employment records) | 3 |
|  |  |
| TOTAL | 85 |

**Appendix S8.**


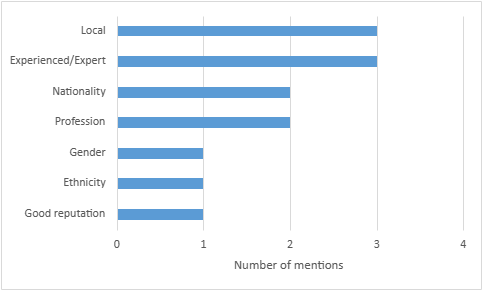


Figure S8. Descriptors applied to research assistants (N = 13 mentions across 24 papers that reported the use of research assistants).

**Appendix S9**

Languages mentioned as spoken at field site (N = 22 papers that mentioned language)

Bahasa Indonesian

Cebuano

Dagaabe

Dzongkha (along with “several” other local languages)

English

Filipino

Gilbertese

Giriama

Gujjari

Hausa

Hindi

Kannada

Kiribati

Kriol

Lao

Malagasy

Makua

Mende

Mongolian

Nepali

Portuguese

Rukiga

Rukonjo

Rutoro

Spanish

Swahili

Sissali

Tagalog (Filipino)

Twi

Vietnamese

Wala
